# Characterizing the resistome of *Escherichia coli* isolated from farmed white-tailed deer (*Odocoileus virginianus*) in Florida, United States

**DOI:** 10.3389/fmicb.2025.1681096

**Published:** 2025-10-23

**Authors:** Austin C. Surphlis, An-Chi Cheng, Morgan C. Metrailer, Andrew P. Bluhm, Treenate Jiranantasak, Frank Tuozzo, Enrique Doster, Jason K. Blackburn, Kwangcheol C. Jeong, Christina Boucher, Juan M. Campos-Krauer, Samantha M. Wisely, Kuttichantran Subramaniam

**Affiliations:** ^1^Department of Infectious Diseases and Immunology, College of Veterinary Medicine, University of Florida, Gainesville, FL, United States; ^2^Emerging Pathogens Institute, University of Florida, Gainesville, FL, United States; ^3^Department of Large Animal Clinical Sciences, College of Veterinary Medicine, University of Florida, Gainesville, FL, United States; ^4^Spatial Epidemiology and Ecology Research Laboratory (SEER Lab), Department of Geography, University of Florida, Gainesville, FL, United States; ^5^VERO Program, Texas A&M University, Canyon, TX, United States; ^6^Department of Animal Sciences, Institute of Food and Agricultural Sciences University of Florida, Gainesville, FL, United States; ^7^Department of Computer and Information Science and Engineering, University of Florida, Gainesville, FL, United States; ^8^Department of Wildlife Ecology and Conservation, Institute of Food and Agricultural Sciences University of Florida, Gainesville, FL, United States

**Keywords:** antimicrobial resistance, resistome, white-tailed deer, *Escherichia coli*, whole genome-sequencing, antimicrobial resistance genes, drug resistance

## Abstract

**Background:**

Antimicrobial resistance (AMR) is a critical public health issue; with many experts suggesting we are already in a post-antibiotic era. The widespread use of antibiotics in agriculture, human, and veterinary medicine influences the spread of antibiotic resistance genes (ARGs) among humans, animals, and the environment. In Florida, white-tailed deer (WTD; *Odocoileus virginianus*) farming plays a vital role in the economy and environment, but the use of antimicrobials in farmed WTD, along with their proximity to urban and agricultural areas, increases the pressure for AMR development. Understanding the resistance patterns in these deer populations is crucial for their health, as well as for wildlife and ecosystems. This research aimed to investigate the resistome of Florida-farmed WTD. *Escherichia coli*, a commonly used indicator bacterium, was chosen to study AMR due to its pathogenicity and ease of culture.

**Methods:**

Samples from various tissues were collected during necropsy. *Escherichia coli* was isolated and cultured, and whole-genome sequencing was performed using a high-throughput NovaSeq platform. The AMR++ v 3.0 pipeline and ResistoXplorer tool were employed for data normalization and analysis. Antimicrobial susceptibility testing of the *E. coli* isolates was conducted using the Kirby-Bauer disk diffusion method on Mueller-Hinton agar, based on the guidelines and recommendations in the CLSI VET01S.

**Results:**

A total of 362 unique ARGs were identified, conferring resistance to 12 antimicrobial classes via 19 mechanisms. The most abundant classes were ß-lactams, multi-drug resistance, and bacitracin. Antimicrobial susceptibility testing showed that 30% of *E. coli* isolates were resistant to at least one drug under aerobic conditions, while 68% were resistant under anaerobic conditions. Moreover, 15% of isolates displayed multi-drug resistance in both conditions. The study also compared genotypic and phenotypic AMR profiles using kappa, revealing good to very good agreement for several drugs.

**Conclusion:**

This is the first study to characterize the resistome of farmed WTD in Florida, providing valuable data for better management of antimicrobial use in these populations.

## 1 Introduction

**Figure 1:**
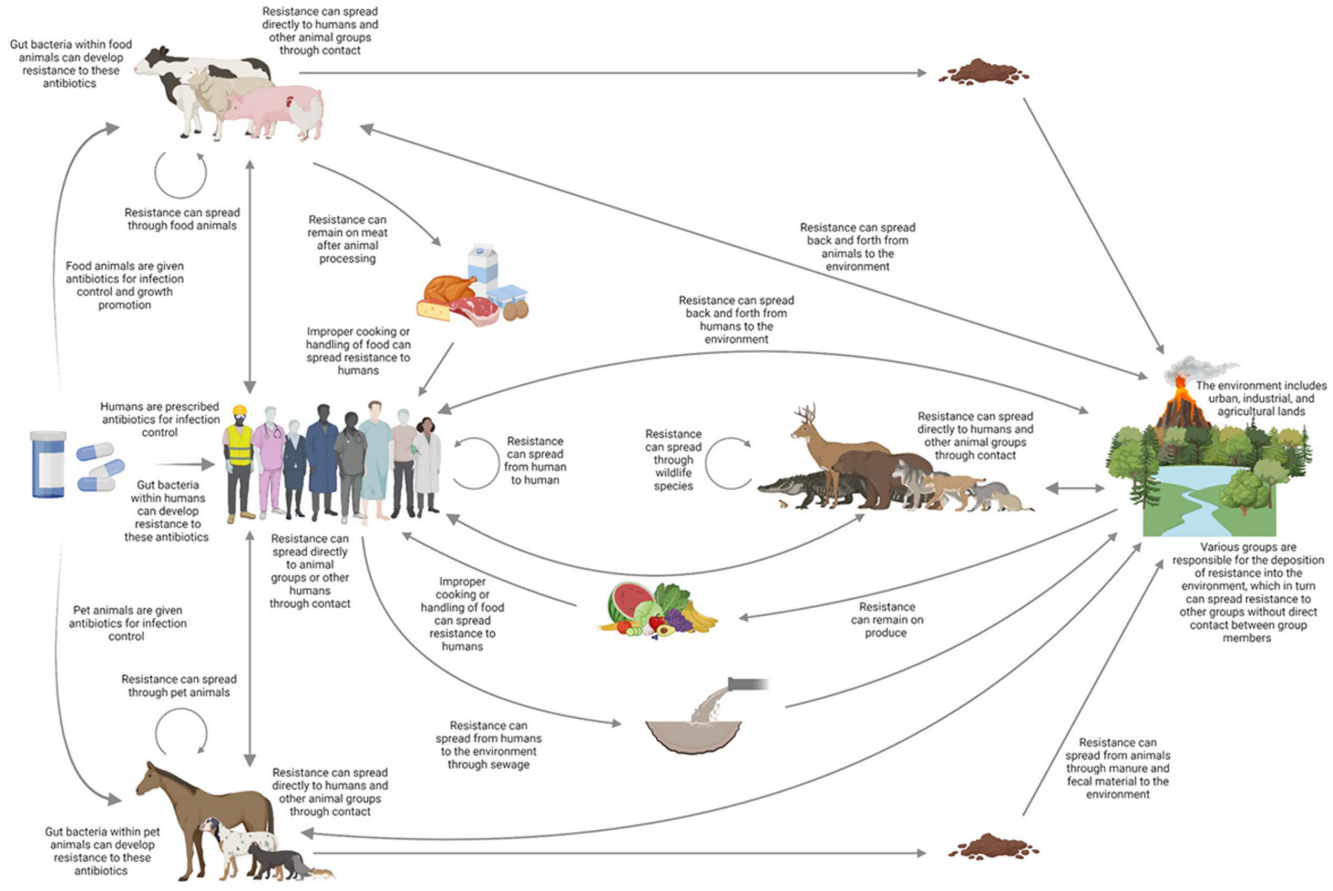
Routes of AMR may be transmitted through various direct and indirect routes to humans, livestock, companion animals, the environment, and wildlife shown by arrows. The overall effectiveness of these channels will vary significantly depending on the type of microbe and resistance mechanism, as well as the environment and location. Adapted from a diagram of the intricate process presented by Woolhouse and Ward (2013).

The resistome, first conceptualized by D'Costa et al. (2006) in response to the discovery of antimicrobial resistance (AMR) traits in soil bacteria, encompasses “the collection of all antibiotic resistance genes and their precursors in both pathogenic and non-pathogenic bacteria” (Wright, 2007). As neither antimicrobial resistance genes (ARGs) nor their bacterial hosts are confined by physical or operational boundaries, this broad definition underscores AMR’s significance as a global public health concern (Figure 1). Resistome characterization is critical to understanding ARG distribution, variation, and abundance across hosts and habitats. Early studies relied on culture-dependent techniques (Benveniste and Davies, 1973), but next-generation sequencing (NGS) and whole-genome sequencing (WGS) have since revolutionized resistome analysis (Ellington et al., 2017; Witney et al., 2016; Crofts et al., 2017). While metagenomics offers a powerful analytical tool, challenges such as sensitivity limitations, sequencing depth, and host DNA contamination persist (Abayasekara et al., 2017; Zhou et al., 2015). In contrast, WGS-based approaches for AMR analysis also present several limitations, including the need for pure cultures, an incomplete representation of the resistome, difficulties in detecting mobile genetic elements (e.g., plasmids and transposons) due to short-read sequencing, gaps in predicting phenotypic resistance, and the labor- and time-intensive nature of the process (Sadovska et al., 2024; Zwe et al., 2020; Weinmaier et al., 2023; Tagami et al., 2024; McDermott et al., 2016; Fauzia et al., 2023).

The prevalence of AMR bacteria is increasingly common in companion and food-producing animals, which is likely linked to the selective pressure from prolonged antimicrobial use in livestock production, veterinary care, and direct human contact (Agersø and Aarestrup, 2013; Durso and Cook, 2014). Peridomestic wildlife comprises species that have adapted to human-modified environments and persist in close proximity to humans, including those deliberately introduced for consumption (Haider et al., 2020). These animals often serve as reservoirs of antimicrobial-resistant microorganisms, although resistance prevalence varies across taxa and geographic regions (Arnold et al., 2016). This variation is influenced by several factors, including environmental contamination primarily from anthropogenic sources, which drives acquired resistance in wildlife (Vittecoq et al., 2016). Although clinically used antimicrobials are not typically found in wild environments, AMR can still occur. Resistance factors have been identified in remote and historically isolated settings, including ancient permafrost layers dating back 30,000 years (D'Costa et al., 2011), secluded cave ecosystems (Bhullar et al., 2012), and even frozen human remains from the Copper Age (Lugli et al., 2017). Nevertheless, numerous environmental exposure pathways—such as manure, wastewater, and pollution from areas with intense anthropogenic activity—can contribute to the selection and dissemination of resistance (Martinez, 2009).

For example, cervids often forage on croplands fertilized with compost, a known hotspot for ARGs, AMR bacteria, and antimicrobials (Rogers et al., 2018; Lima et al., 2020). Additionally, freshwater sources, critical to wildlife hydration, are similarly vulnerable to antimicrobial contamination from sewage and agricultural runoff (Zhu et al., 2017; Zhou et al., 2018; Cacace et al., 2019; Nnadozie and Odume, 2019). Evidence also supports the significant role migratory birds play in the spread of AMR (Pinto et al., 2010; Hasan et al., 2014). Furthermore, the host diet can influence gut microbiota dynamics, affecting the prevalence of AMR microorganisms among commensal bacteria (Williams et al., 2011). A predominantly herbivorous diet may account for the generally low levels of AMR observed in many wildlife species that graze on vegetation, in contrast to the higher levels seen in species with omnivorous or carnivorous diets (Vittecoq et al., 2016).

White-tailed deer (WTD; *Odocoileus virginianus*), widely distributed across the Americas, inhabit diverse environments, from natural ecosystems to urban areas (Ballash et al., 2022). As a keystone species, WTD influence food web dynamics, notably as prey for many species including the endangered Florida panther (Boughton et al., 2020). Cervid hunting and farming in the U. S. contribute significantly to both the economy and ecosystem management (Anderson et al., 2017; Brooks et al., 2015; Lantz, 1908; Hewitt, 2015; Conover, 2011). Deer hunting generates over $13 billion annually, supporting 209,000 jobs in the southeastern U. S. alone (National Deer Association, 2022). Ecologically, hunting helps regulate deer populations, mitigating overbrowsing that disrupts understory vegetation (Marquis and Brenneman, 1981; Warren, 1991). WTD, the most commonly farmed cervid, also provide environmental benefits by thriving on brushlands unsuitable for cattle or horses (Lantz, 1908).

Cervid farming, which is currently expanding in Florida, is situated in a state where AMR bacteria are prevalent in hospitals, livestock, companion animals, and wildlife (Sy-Trias, 2023; Gutierrez et al., 2020; Giguère et al., 2010; White and Forrester, 1979; Greig et al., 2007). However, antimicrobial use in farmed WTD, coupled with proximity to urban and agricultural areas, may drive AMR development. While the implications for Florida’s cervid farming remain unclear, AMR-associated morbidity and mortality could pose risks for WTD farmers. Assessing resistance patterns is crucial for managing cervid health and preserving ecosystem integrity.

However, the AMR in Florida farmed WTD is unknown. To date, AMR research in cervids has primarily focused on antimicrobial susceptibility of indicator bacteria such as *Escherichia coli* and *Enterococcus* spp., as well as pathogens like *Campylobacter* spp. and *Salmonella* spp. (Dias et al., 2015; Plaza-Rodríguez et al., 2021; Turchi et al., 2019). High-throughput metagenomic sequencing enables comprehensive ARG profiling, yet studies on ARGs in cervids remain scarce (Rogers et al., 2018). Hence, this study aims to characterize the genotypic and phenotypic profiles of ARGs in *E. coli* isolates from these cervid populations using high-throughput sequencing, ARG annotation through publicly available databases, and antimicrobial susceptibility testing.

*E. coli* is widely recognized as a valuable indicator of AMR in wildlife and environmental monitoring (Anjum et al., 2021). Studies have demonstrated a significant correlation between the presence of AMR in *E. coli* and the detection of resistant pathogenic strains within the same sample (Nyirabahizi et al., 2020). Due to its role as an indicator organism, strain-specific pathogenicity, and ease of culture (Anjum et al., 2021), *E. coli* was selected in this study for AMR characterization from farmed WTD in Florida. In addition, to address the limitations of metagenomic approaches—namely sensitivity constraints, limited sequencing depth, and host DNA contamination—this study employs WGS of *E. coli*.

## 2 Materials and methods

### 2.1 Sample collection and initial processing

A total of 60 tissue samples—including kidney, lung, liver, and heart—were collected during necropsies of 51 individual WTD. Of these, 33 samples were obtained from 30 clinically ill deer, which were either euthanized (2/30) or found dead (28/30). The 2 animals were euthanized by the farm owner due to bacterial pneumonia. When clinical signs such as lameness or upper respiratory illness are observed, owners may choose to initiate treatment, typically with antibiotics or supportive care, or proceed with euthanasia. Because deer, as prey animals, often mask signs of illness until advanced stages, treatment outcomes are frequently limited. Some owners attempt treatment until the animal is no longer viable, whereas others elect euthanasia earlier to reduce suffering. Consequently, there is no standardized threshold for this decision, and practices vary on a case-by-case basis. The remaining 27 samples were collected from deer that were found dead without prior clinical signs and necropsied within 24 h of death. These samples were gathered from 16 counties in Florida, U. S., between September 16, 2020, and November 14, 2022. The organs were grossly examined, and tissue was collected and placed in 5 mL sterile Eppendorf snap cap tubes (Thermo Fisher Scientific) in a cooler with ice packs while in the field and subsequently transferred to a 4 °C refrigerator. The WTD tissue samples were submitted to the Clinical Microbiology Laboratory at the University of Florida (UF) College of Veterinary Medicine (CVM) for aerobic or anaerobic bacterial culture, depending on the sample type and diagnostic suspicion. CNA agar (Columbia Nalidixic Acid) is a selective medium designed for the isolation of Gram-positive bacteria. Since *E. coli* is a Gram-negative organism and the primary target of this study, no growth was expected or observed on CNA agar. The choice between aerobic and anaerobic culture conditions was guided by both sample type and the clinical or diagnostic context. For example, lung tissues were generally cultured under aerobic conditions due to their natural exposure to oxygen, whereas pus or lesion specimens were more often cultured anaerobically, reflecting their origin in enclosed or oxygen-limited environments. In addition, the suspected characteristics of the pathogen influenced this decision. Importantly, as a facultative anaerobe, *E. coli* was successfully isolated under both aerobic and anaerobic conditions. This work was approved by the UF Institutional Animal Care and Use Committee (IACUC Protocol Numbers 201609390 and 201909390).

### 2.2 Bacterial isolation and identification

Tissue samples were cultured specimens were cultured in their native form, directly incubated on differential media, including MacConkey agar (MAC), Columbia Nalidixic Acid agar (CNA), chocolate agar (CHOC), and general blood agar plates for bacterial isolation. No growth was observed on CNA agar. MALDI-TOF (MALDI Biotyper sirius one System from Bruker) was used for the identification of bacteria from the MAC, CHOC, and blood agar plates. Single colonies were then picked from *E. coli* positive plates and placed into CryoSavers tubes consisting of Brucella broth and 10% glycerol (Hardy Diagnostics) utilizing disposable inoculation wands (Thermo Fisher Scientific). The CryoSavers tubes were then stored in a −80 °C freezer until they were reinoculated into brain heart infusion (BHI) (DB Difco 237500) broth at 37 °C with shaking overnight at 220 revolutions per minute (RPM) for WGS and antimicrobial susceptibility testing (AST).

### 2.3 DNA extraction and whole genome sequencing

One milliliter of the overnight culture was pelleted at 10,000× relative centrifugal force (RCF) for one minute. Then, the genomic DNA of the *E. coli* was extracted using the Wizard Genomic DNA Purification Kit (Promega #A1120), according to the manufacturer’s protocol. The sequencing libraries were prepared using the Illumina ILMN DNA LP Kit (#20060059) and IDT for Illumina DNA/RNA UD Indexes Set A (#20027213). The DNA libraries were then sequenced at the UF Interdisciplinary Center for Biotechnology Research (ICBR) NextGen DNA Sequencing core (RRID: SCR_019152) using an Illumina NovaSeq sequencer with 2 × 150 cycle S4 kit (Bruinsma et al., 2018). The *E. coli* isolates were categorized into distinct phylogroups using the EZclermont web-based tool, allowing for a clear illustration of the collection’s diversity (Waters et al., 2020).

### 2.4 Data management and resistance gene analyses

To characterize the resistome of the *E. coli* isolates, the AMR++ Pipeline (version 3.0) (Bonin et al., 2023) was utilized. The AMR++ Pipeline trims the raw paired-end reads (FASTQ) for quality control and then aligns them to approximately 9,000 hand-curated ARGs from the MEGARes database 3.0 (https://www.meglab.org) using Burrows-Wheeler-Aligner (BWA) to produce Sequence Alignment/Map (SAM) format (Lakin et al., 2017). The Single Nucleotide Polymorphism (SNP) confirmation and “deduped” functions of the pipeline were implemented to confirm SNPs and deduplicate counts. Sequences from public databases that have been published and represent distinct accession numbers were referred to as individual ARGs. The main function of the structural genes in the MEGARes database is regulatory activity (e.g., efflux system), even though they are typically encoded on the chromosome and do not dictate phenotypic resistance. Three levels of hierarchical classification were established by aggregating ARGs: class (e.g., phenicols), mechanism (e.g., Phenicol resistance MFS efflux pumps), and group (e.g., *floR*) (Lakin et al., 2017). In addition to maintaining reasonable biological categories throughout the database, the group annotation provides insights into the primary gene classification while preserving nucleotide arrangements. Only ARGs that were more than 80 percent covered by sample reads and for which a single nucleotide polymorphism (SNP) did not confer resistance were taken into consideration for further examination.

ResistoXplorer (https://www.resistoxplorer.no) was implemented for data normalization, analysis, and visualization (Dhariwal et al., 2021). The low-count and variance filters of the web-based tool were used to filter poor quality or non-informative ARGs to improve downstream comparative analyses. The low count filters were adjusted so that the software only included features present in at least one count and were prevalent in at least 10% of samples. The low variance filter was modified so that zero percentage variance was removed, and the calculation was based on the standard deviation. The Cumulative Sum Scaling (CSS) (Paulson et al., 2013) function was applied to remove any possible sampling or sequencing biases and to normalize the ARG counts before converting for relative abundance and core resistome analyses. CSS is similar to total sum scaling (TSS), which calculates the ratio of the read count for each ARG compared to the total read count per sample. However, to reduce the impact of highly abundant variable genes, the denominator is calculated by summing the total read counts, starting with low-abundance genes and continuing up to a predefined threshold. In a comparative analysis of nine normalization techniques for count data, CSS emerged as one of the most effective methods for handling large metagenomic datasets (Pereira et al., 2018). The relative abundance and core resistome matrices for different levels (e.g., class and mechanism) were then produced after CSS counts were aggregated for varying resistance levels (e.g., class and mechanism). These matrices were used to generate the plots in RStudio (R version 4.0.3), which illustrate the class and mechanism level relative abundance as well as the core resistome for all *E. coli* isolates.

### 2.5 Statistical analysis: genotype

Alpha diversity of the resistome was assessed using the Shannon diversity index, which accounts for both richness and evenness but does not capture compositional differences and is not normally distributed. Shannon indices were calculated for each sample across resistance profiles classified at the class, mechanism, group, and gene levels. To evaluate the influence of metadata variables—including phylogroup, isolate origin, and farm—on resistome diversity, analysis of variance (ANOVA) was applied, acknowledging its assumptions of normality and homoscedasticity. This approach facilitated the identification of potential associations between environmental or biological factors and the complexity of antimicrobial resistance patterns.

### 2.6 Antimicrobial susceptibility testing

Antimicrobial susceptibility testing of the *E. coli* isolates was conducted using the Kirby-Bauer disk diffusion method on Mueller-Hinton agar, based on the guidelines and recommendations in the CLSI VET01S (CLSI, 2024). Thirteen antimicrobials were selected for testing based on their frequent use in Florida deer farms and their relevance in both human and veterinary medicine. These include: ampicillin (AMP, 10 μg) (Becton Dickinson and Company, Sparks, MD, United States), penicillin (PEN, 10 μg) (Becton Dickinson and Company, Sparks, MD, United States), ceftiofur (CEFT, 30 μg) (Becton Dickinson and Company, Sparks, MD, United States), tetracycline (TET, 30 μg) (Becton Dickinson and Company, Sparks, MD, United States), oxytetracycline (OXY, 30 μg) (Becton Dickinson and Company, Sparks, MD, United States), gentamicin (GENT, 10 μg) (Becton Dickinson and Company, Sparks, MD, United States), neomycin (NEOM, 30 μg) (Becton Dickinson and Company, Sparks, MD, United States), NUFLOR® (florfenicol, FLU, 300 mg ml^−1^) (Merck & Co., Inc., Rahway, NJ, United States), Resflor Gold® (florfenicol + flunixin meglumine, FF, 300/16.5 mg ml^−1^) (Merck & Co., Inc., Rahway, NJ, United States), trimethoprim-sulfamethoxazole (SXT, 1.25/23.7 μg) (Becton Dickinson and Company, Sparks, MD, United States), enrofloxacin (ENRO, 5 μg) (Becton Dickinson and Company, Sparks, MD, United States), Draxxin® (tulathromycin, TUL, 100 mg ml^−1^) (Zoetis Inc. Kalamazoo, MI, United States), and ZACTRAN® (gamithromycin, GAM, 150 mg ml^−1^) (Boehringer Ingelheim Animal Health United States Inc., Duluth, GA, United States). Fifteen microliters of the commercial antimicrobials—NUFLOR®, Resflor Gold®, Draxxin®, ZACTRAN®—were applied to blank disks and left overnight covered in petri dishes while in a biosafety cabinet. The final antimicrobial amounts per disk were 4.5 μg, 4.5/0.425 μg, 1.5 μg, and 2.25 μg, respectively. Susceptibility results were interpreted using zone of inhibition breakpoints established by the CLSI for AMP, CEFT, TET, OXY, GENT, and SXT, and by the NCCLS for NEOM. Multi-drug resistance was defined as resistance to at least three antimicrobial agents as described by Begum et al. (2018).

The isolates stored in CryoSavers tubes, consisting of Brucella broth and 10% glycerol (Hardy Diagnostics), at −80 °C were reinoculated into brain heart infusion (BHI) (BD Difco 237500; Becton, Dickinson and Company) broth at 37 °C with shaking overnight at 220 revolutions per minute (RPM). The culture was adjusted to an optical density (OD) of 0.1 (approximately 0.5 McFarland standards) in BHI. Sterile swabs were dipped into the adjusted bacterial culture and gently rolled on the inside wall of the tube to squeeze out any excess culture. The entire surface of the Mueller-Hinton agar plate was swabbed in three directions, with the plate being rotated 60° after each pass to ensure even distribution and create a uniform bacterial lawn. The plates were then allowed to dry for no more than 15 min. A Sensi-Disc Dispenser (BD 260660; Becton, Dickinson and Company) was used to place 8 disks, while the remaining 5 were applied manually using sterile forceps. A minimum distance of 24 mm between the centers of adjacent disks and 15 mm from the edge of the plate was maintained. Each disk was gently pressed to ensure firm contact with the agar surface. The plates were inverted and incubated at 37 °C for 16–18 h under aerobic or anaerobic conditions. Following incubation, the diameter of the clear zone around each antimicrobial disk was measured in millimeters. To account for potential irregularities in the shape of the zone of inhibition (ZOI), measurements were taken at three different points along the edge and then averaged. The CLSI and NCCLS breakpoints were applied to classify each isolate as resistant, intermediate, or susceptible.

### 2.7 Statistical analysis: phenotype

A generalized linear model (GLM) with a Poisson distribution and log link function was used to assess the effects of phylogroup, isolate, and farm on phenotypic resistance counts. The GLM was constructed using phenotypic resistance counts as the dependent variable and the tested factors—phylogroup, isolate, and farm—as independent variables. This approach allowed for the assessment of potential associations between these factors and the observed resistance phenotypes while accounting for variability within the data. Statistical significance was determined by calculating *p*-values for each factor, with a threshold of 0.05 indicating significance.

### 2.8 Correlation among antimicrobial resistant genotypes and phenotypes

The resistant genotype of the *E. coli* isolates was identified through whole genome sequencing, and the use of the AMR++ pipeline to align the sequencing reads to a curated ARG database. A comprehensive workflow for genotype and phenotype determination of the *E. coli* isolates is outlined in the above sections. Cohen’s kappa statistics were used to assess the agreement between genotype and phenotype. The presence of the *cmy*, *ctx*, or *tem* gene groups was classified as a resistant genotype for AMP and CEFT, while the *tetA*, *tetB*, or *tetD* gene groups were considered a resistant genotype for TET and OXY. For GENT and NEOM, a resistant genotype was determined by the existence of the *aac3* gene group, and for FLU, the *floR* or *cmlA*, gene groups. A resistant genotype to ENRO was linked to the *qnrS* gene group. Due to the complex interaction of SXT, which inhibits two pathways in folate synthesis, the simultaneous presence of the *sulII*, *sulIII*, and *dfrA* gene groups was considered a resistant genotype (Projan, 2002). The ZOI breakpoints for *Enterobacterales*, as outlined by CLSI and NCCLS, were used to classify phenotypic resistance. For statistical purposes, isolates in the intermediate category were treated as susceptible (Schwan et al., 2021). Kappa coefficient (*κ*) values were interpreted as follows: <0.2 = poor, 0.21–0.4 = fair, 0.41–0.6 = moderate, 0.61–0.8 = good, and 0.81–1.0 = very good agreement (Azen and Walker, 2021). A two-sided *z*-test with a *p*-value < 0.05 was considered statistically significant.

## 3 Results

### 3.1 Animal information and phylogroup

The 60 *E. coli* strains were collected from tissue samples obtained during the necropsy of 51 individual WTD. The bacteria were initially isolated, and identified using MALDI-TOF, followed by DNA extraction and whole genome sequencing. Following sequencing, the *E. coli* isolates were typed by phylogroup to assess their diversity, employing methods described by Metrailer et al. (2024). The isolates were grouped into seven distinct phylotypes: A, B1, B2, C, D, E, and F (Table 1). Of the 60 isolates, 75% (45/60) grouped into phylogroup B1, while 11.67% (7/60), 0.05% (3/60), 0.03% (2/60), 0.01% (1/60), 0.01% (1/60), 0.01% (1/60), grouped into phylogroups A, D, E, B2, C, and F, respectively.

**Table 1:** Typing information for animals and *E. coli* isolates collected from farmed WTD in Florida between September 2020 and November 2022 (*n* = 60).

Sample	Necropsy date	County	Phylotype	Phenotype	Genotype
S1	19-Sep-20	Gadsden	A	0	5
S2	8-Oct-20	Lafayette	B1	4	7
S3	8-Oct-20	Lafayette	B1	4	6
S5	23-Oct-20	Calhoun	B1	2	6
S6	23-Oct-20	Calhoun	B1	2	6
S4	23-Oct-20	Calhoun	B1	0	6
S7	2-Nov-20	Calhoun	B1	2	9
S8	23-Nov-20	Liberty	B1	0	5
S9	4-Dec-20	Liberty	A	0	4
S10	28-Dec-20	Marion	B1	0	4
S11	12-Jan-21	Marion	A	0	3
S12	21-Jan-21	Calhoun	B1	0	4
S13	21-Jan-21	Calhoun	B1	0	4
S14	25-Jan-21	Okeechobee	E	0	4
S15	29-Jan-21	Calhoun	B1	0	5
S16	29-Jan-21	Calhoun	B1	0	5
S17	19-Feb-21	Jefferson	D	0	5
S18	19-Feb-21	Jefferson	D	0	5
S19	22-Feb-21	Gadsden	E	0	5
S20	13-Mar-21	Marion	B1	0	4
S21	13-Mar-21	Marion	B1	0	4
S22	13-Mar-21	Marion	A	0	3
S35	28-Sep-21	Gadsden	B1	2	8
S23	11-Jun-21	Marion	B1	3	9
S24	11-Jun-21	Marion	B1	3	9
S25	11-Jun-21	Marion	B1	2	6
S26	14-Jul-21	Columbia	B1	0	4
S28	29-Jul-21	Gadsden	D	0	5
S27	29-Jul-21	Gadsden	C	0	5
S29	27-Aug-21	Hillsborough	B1	0	4
S30	28-Aug-21	Liberty	B1	0	4
S31	30-Aug-21	Liberty	A	0	4
S32	1-Sep-21	Nassau	B1	0	11
S33	4-Sep-21	Nassau	B1	0	5
S34	27-Sep-21	Marion	B1	6	9
S37	29-Sep-21	Sumter	B2	0	5
S36	29-Sep-21	Sumter	B1	0	4
S38	8-Oct-21	Liberty	A	0	5
S40	20-Oct-21	Liberty	B1	0	4
S41	9-Nov-21	Gadsden	B1	0	5
S42	23-Nov-21	Gadsden	B1	2	6
S43	7-Mar-22	Gadsden	B1	4	9
S44	21-Mar-22	Liberty	F	2	5
S45	4-Apr-22	Jackson	B1	0	5
S46	23-May-22	Calhoun	B1	0	5
S47	22-Jul-22	Gadsden	B1	0	5
S48	29-Jul-22	Hernando	B1	0	4
S49	11-Aug-22	Martin	B1	6	8
S50	7-Sep-22	Clay	B1	2	6
S51	13-Sep-22	Hillsborough	B1	0	7
S52	6-Oct-22	Gadsden	B1	6	10
S53	7-Oct-22	Suwannee	B1	6	9
S54	7-Oct-22	Suwannee	B1	2	5
S55	10-Oct-22	Suwannee	B1	0	5
S56	11-Oct-22	Jackson	B1	0	5
S57	12-Oct-22	Suwannee	B1	0	5
S58	14-Oct-22	Suwannee	B1	0	5
S59	15-Oct-22	Suwannee	B1	2	6
S60	18-Oct-22	Suwannee	B1	0	5
S61	14-Nov-22	Jackson	A	0	5

Genotype (number of antimicrobial classes each sample had at least one ARG for). Phenotype (number of drugs each sample was phenotypically resistant to). Phylotype (EzClermont).

### 3.2 Core resistome and abundance profiles

**Figure 2:**
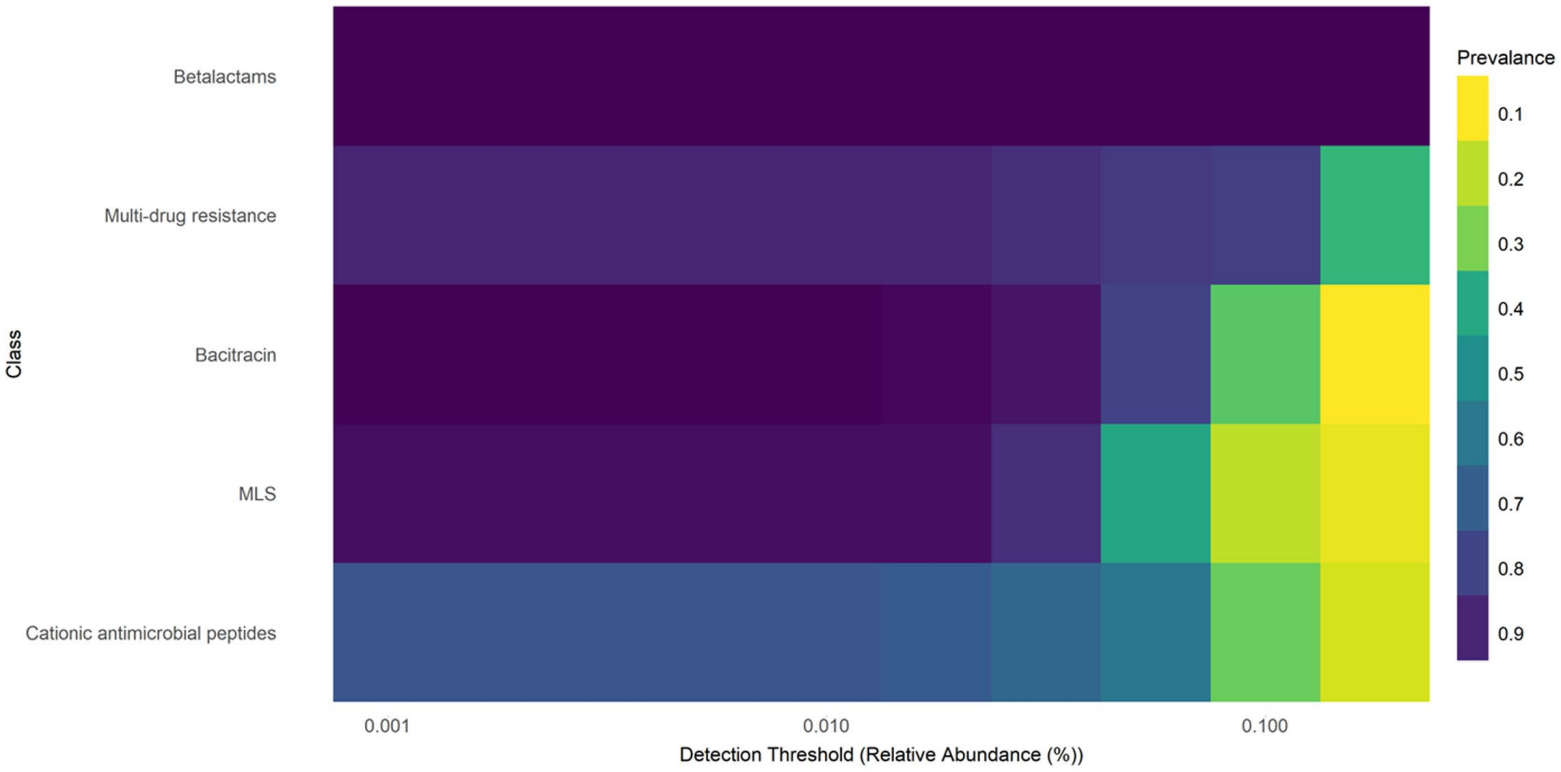
Heatmap of core resistome analysis of *E. coli* isolates from white-tailed deer revealed five classes to be prevalent in at least 10% of the samples. Macrolide, lacosamide, Streptogramin (MLS).

The core resistome refers to the set of ARGs, gene groups, mechanisms, or classes that are consistently detected in a large proportion of a population, exceeding a defined abundance threshold. In *E. coli* isolates from farmed WTD in Florida, the core resistome comprises ARGs associated with five major antimicrobial classes: β-lactams, multidrug resistance (MDR), bacitracin, macrolide-lincosamide-streptogramin (MLS), and cationic antimicrobial peptides (CAP). These ARGs are both abundant and broadly distributed across the sampled population (Figure 2). The heat map of the core resistome illustrates five classes to be prevalent in at least 10% of the samples. For example, the prevalence of the bacitracin class having a relative abundance (or detection threshold) of 0.001%, is 90%. As the detection threshold becomes more stringent, with a relative abundance of at least 0.100%, the prevalence drops to approximately 0.20 (only 20% of samples will contain bacitracin resistance at a relative abundance of 0.100%).

**Figure 3:**
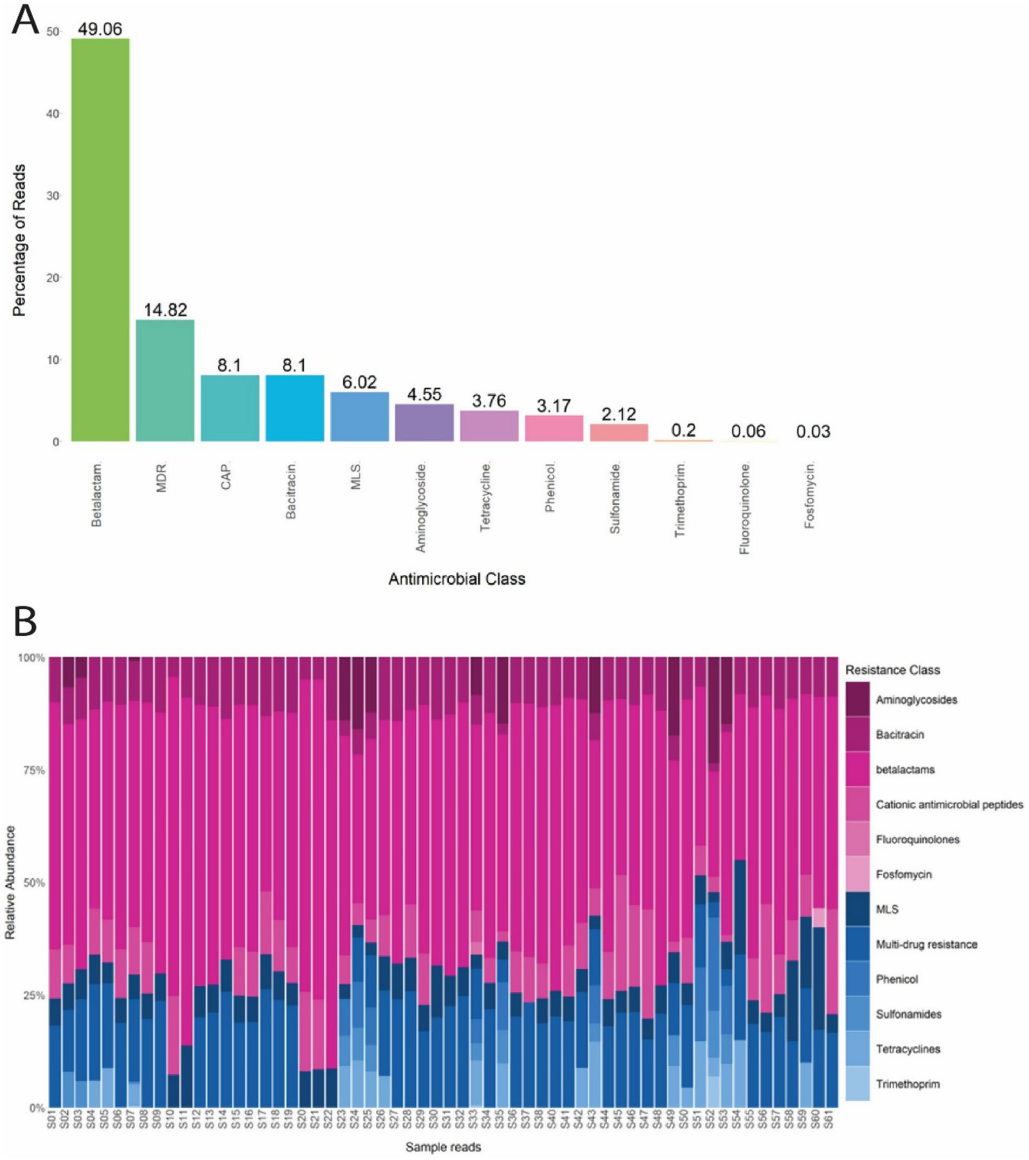
**(A)** Percentage of reads from *E. coli* isolates that confer resistance to each antimicrobial class. **(B)** Normalized relative abundance (percentage) of ARGs in *E. coli* isolates grouped by resistance class for all 60 samples. Multi drug resistance (MDR); Cationic antimicrobial peptides (CAP); Macrolide, lacosamide, Streptogramin (MLS).

Among the 60 *E. coli* samples analyzed, 362 unique ARGs were identified as resistant to 12 antimicrobial classes. Overall, the ARGs identified confer resistance to the ß-lactam (49.06%), MDR (14.82%), CAP (8.1%), bacitracin (8.1%), MLS (6.02%), aminoglycoside (4.55%), tetracycline (3.76%), phenicol (3.17%), sulfonamide (2.12%), trimethoprim (0.2%), fluoroquinolone (0.06%), and fosfomycin (0.03%) antimicrobial class (Figure 3A). ß-lactam and MLS resistance were abundant in every sample tested, and MDR was abundant in 90% (54/60) of the samples tested (Figure 3B).

**Figure 4:**
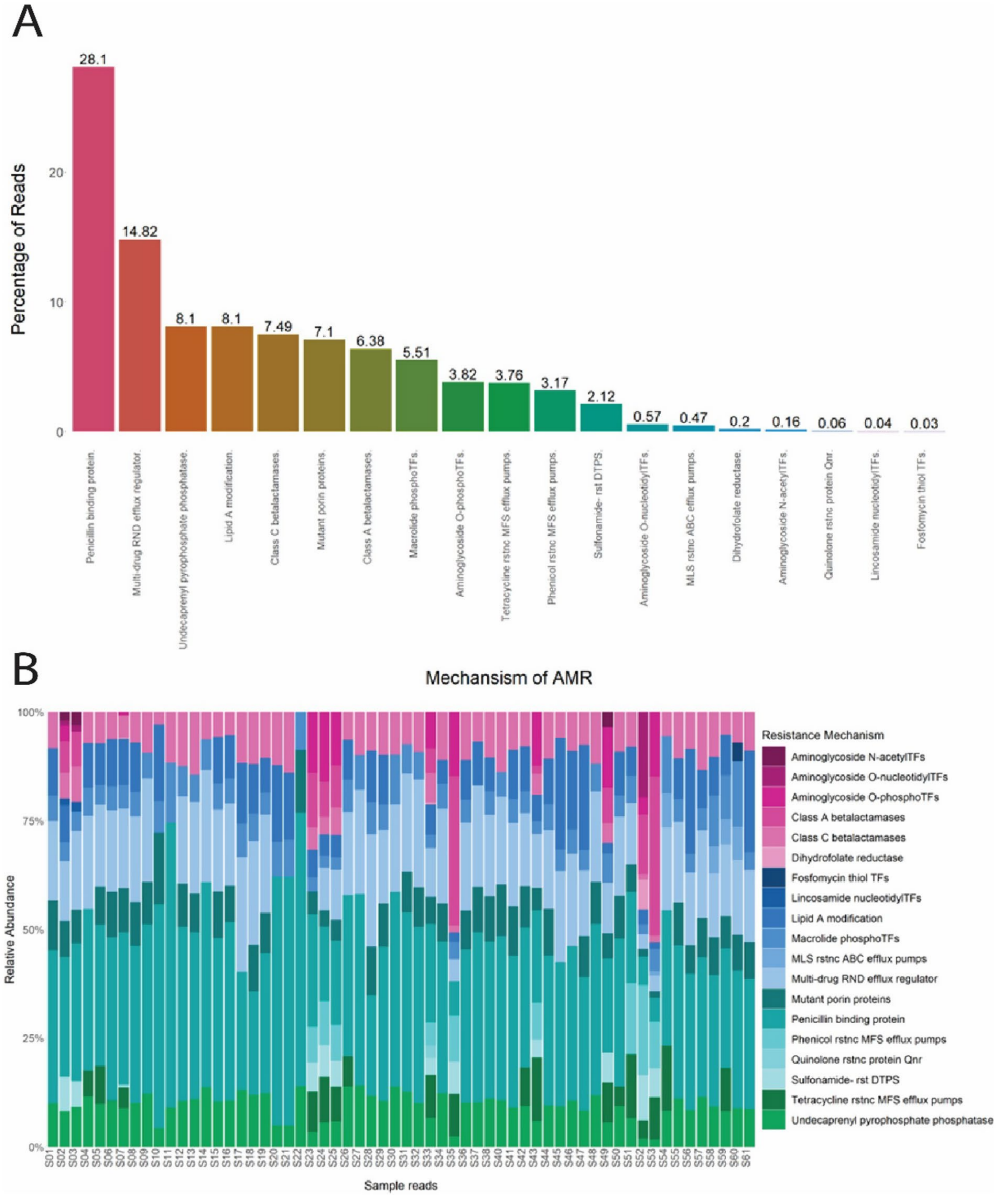
**(A)** Percentage of reads from *E. coli* isolates for each mechanism of antimicrobial resistance. **(B)** Normalized relative abundance (percentage) of ARGs in *E. coli* isolates grouped by resistance mechanism for all 60 samples. N-acetylTFs (N-acetyltransferases); O-nucleotidylTFs (O-nucleotidyltransferases); O-phosphoTFs (O-Phosphotransferases); Transferases (TFs); rstnc (Resistance); ABC (ATP- binding cassette); RND (Resistance-Nodulation-Division); MFS (major facilitator superfamily); rst (resistant); DTPS (dihydropteroate synthase).

The 12 antimicrobial classes confer resistance by 19 mechanisms, including Penicillin binding protein (28.1%), Multi-drug RND efflux regulator (14.82%), Undecaprenyl pyrophosphate phosphatase (8.1%), Lipid A modification (8.1%), Class C β-lactamases (7.49%), Mutant porin proteins (7.1%), Class A β-lactamases (6.38%), Macrolide phosphotransferases (5.51%), Aminoglycoside O-phosphotransferases (3.82%), Tetracycline resistance MFS efflux pumps (3.76%), Phenicol resistance MFS efflux pumps (3.17%), and Sulfonamide- resistant dihydropteroate synthase (2.12%). The remaining seven mechanisms had an overall abundance of less than 1 % and are summarized in Figure 4A. Penicillin binding protein was abundant in every sample tested and mechanisms associated with MLS resistance and MDR were observed in 98.3% (59/60) and 90% (54/60) of samples, respectively (Figure 4B).

**Figure 5:**
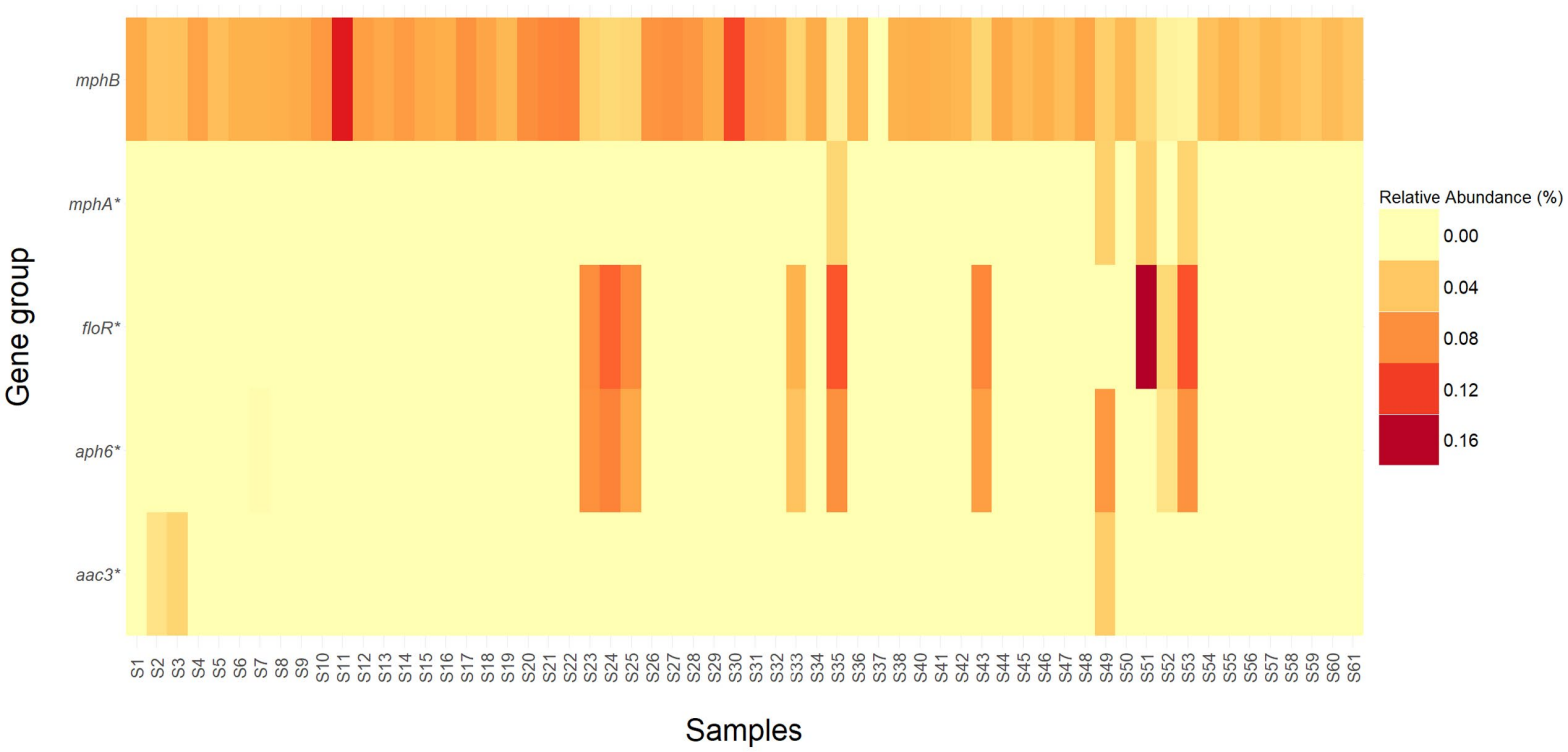
Heatmap representing the relative abundance (percentage) of high-risk ARG groups (Rank 1) detected in each sample. ARG groups *mphA*, *floR*, *aph6*, and *aac3* contain genes that are classified in different risk levels and are denoted by an *.

In addition to the class and mechanism abundance profiles, various level 1 high-risk AMR genes were discovered in relatively high abundances in the current study (Figure 5). A total of five ARG groups that are considered “present hazards” (Zhang et al., 2021) including *aac3*, *aph6*, *floR*, *mphA*, and *mphB* were found in the *E. coli* isolated from the WTD samples. ARG groups: *aac3*, *aph6*, *floR*, *mphA* also contain genes in multiple categories (e.g., Level 3, Level 4, and unassigned). Among the rank 1 ARGs, *mphB* was identified in over 98% (59/60) of the samples tested. Notably, samples S11 and S30 exhibited the highest relative abundances, with *mphB* representing 13.8% (551/527233) and 11.5% (229/52733) of the total reads, respectively. Additionally, *floR,* which is known to confer resistance to chloramphenicol and florfenicol (White et al., 2000), showed a relative abundance of 16.3% (4,122/32,389), the highest percentage among all samples and ARG groups.

Alpha diversity analyses were conducted using analysis of variance (ANOVA) with the Shannon diversity index to assess whether any metadata factors influenced resistance profile levels. Prior to conducting the ANOVA, assumptions of normality and homogeneity of variance were verified. No statistically significant differences were observed between the levels of resistance profiles (class, mechanism, group, and gene) across the experimental factors (region, farm, and phylogroup) (*p*-value > 0.05).

### 3.3 Antimicrobial susceptibility testing

**Figure 6:**
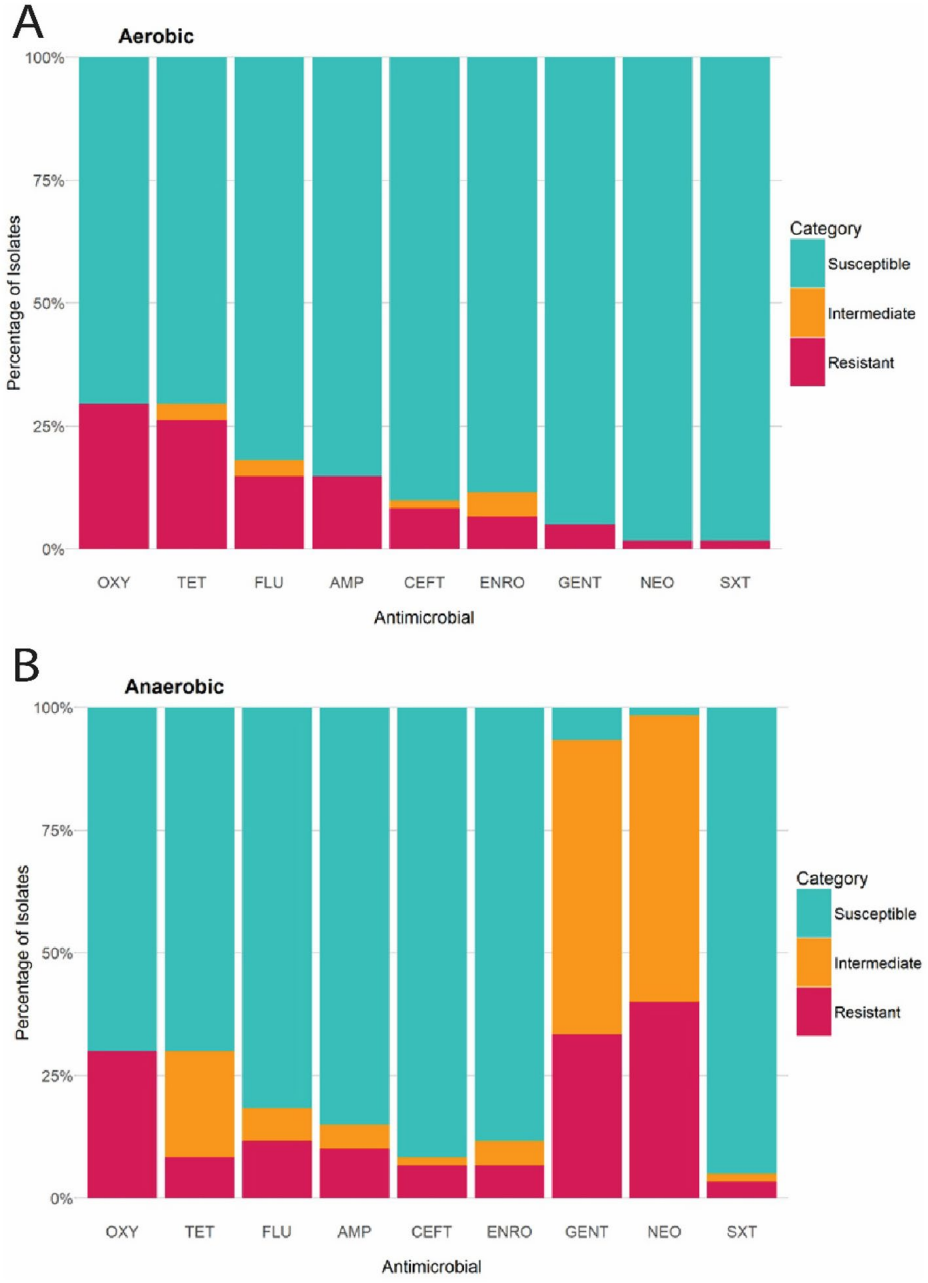
**(A)** Bar chart illustrating the percentage of *E. coli* isolates from WTD classified as Susceptible, Intermediate, or Resistant to each antimicrobial compound, according to CLSI breakpoints under aerobic conditions. **(B)** Bar chart illustrating percentage of *E. coli* isolates from WTD classified as Susceptible, Intermediate, or Resistant to each antimicrobial compound, according to CLSI breakpoints under aerobic conditions. AMP (Ampicillin); CEFT (Ceftiofur); FLU (Florfenicol); NEO (Neomycin); GENT (Gentamicin); ENRO (Enrofloxacin); TET (Tetracycline); OXY (Oxytetracycline); SXT (Trimethoprim–sulfamethoxazole).

**Figure 7:**
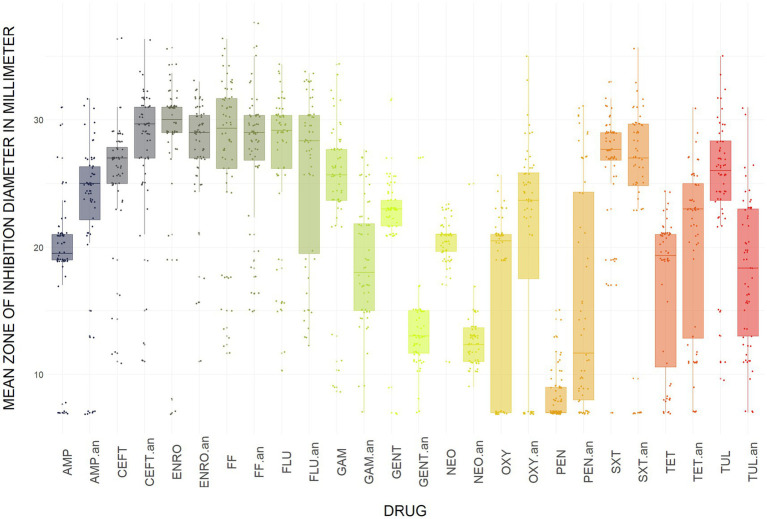
Boxplots and individual points representing the mean zone of inhibition (in millimeters) for *E. coli* isolates from WTD, grouped by antimicrobial agent under aerobic and anaerobic conditions. AMP (Ampicillin); CEFT (Ceftiofur); FLU (Florfenicol); NEO (Neomycin); GENT (Gentamicin); ENRO (Enrofloxacin); TET (Tetracycline); OXY (Oxytetracycline); SXT (Trimethoprim–sulfamethoxazole); FF (Florfenicol + Flunixin meglumine); GAM (Gamithromycin); PEN (Penicillin); TUL (Tulathromycin). “an” indicates anaerobic condition.

**Figure 8:**
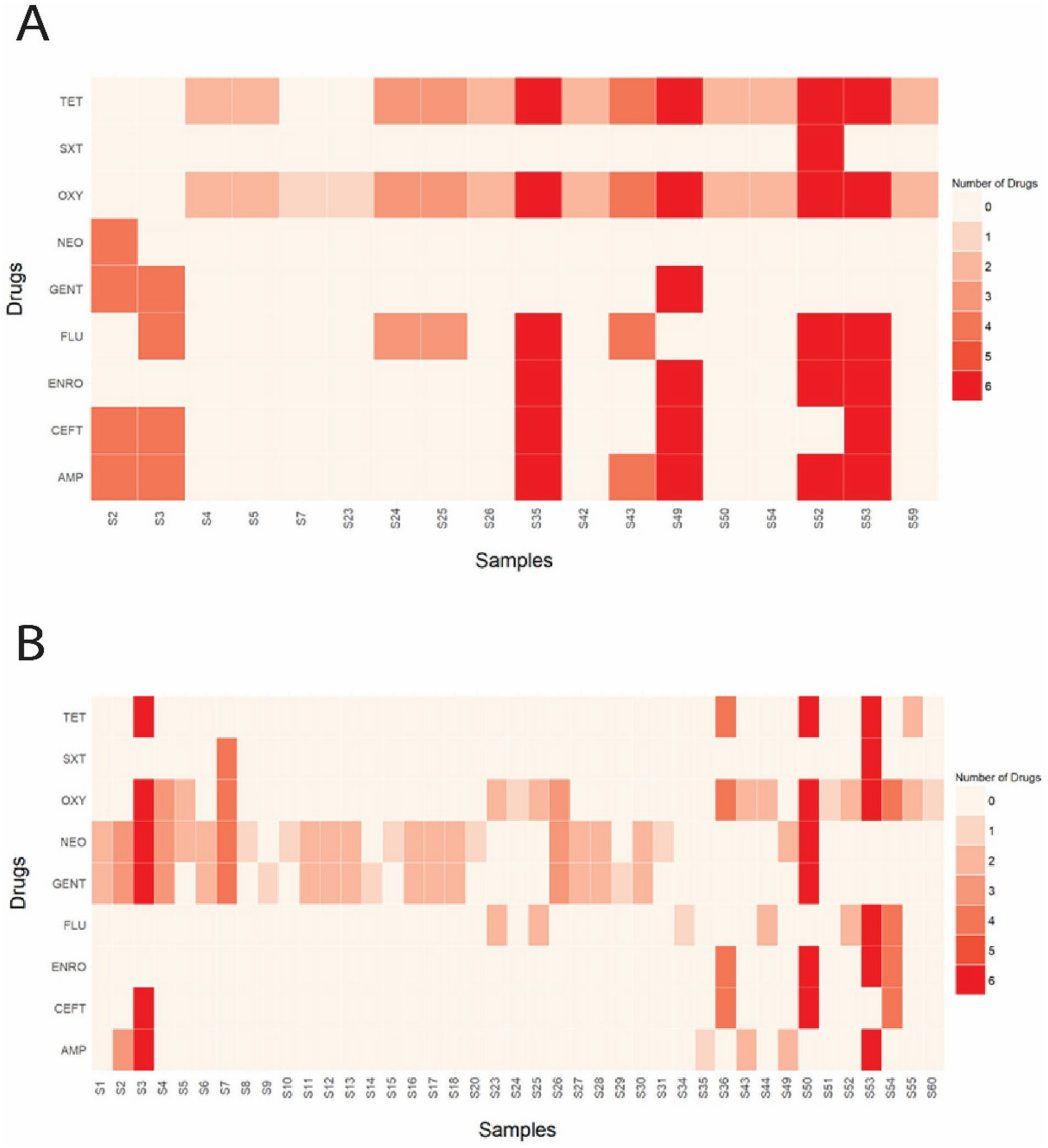
**(A)** Heatmap illustrating the total number and specific drug(s) to which each sample displayed phenotypic resistance under aerobic conditions. **(B)** Heatmap illustrating the total number and specific drug(s) to which each sample displayed phenotypic resistance under anaerobic conditions. AMP (Ampicillin); CEFT (Ceftiofur); FLU (Florfenicol); NEO (Neomycin); GENT (Gentamicin); ENRO (Enrofloxacin); TET (Tetracycline); OXY (Oxytetracycline); SXT (Trimethoprim–sulfamethoxazole).

The Kirby-Bauer disk diffusion method was conducted on 60 *E. coli* isolates from farmed WTD in Florida. The ZOI for each antimicrobial agent varied between aerobic and anaerobic conditions. Under aerobic conditions, the following resistance rates were observed: 14.75% of the isolates were resistant to AMP (Ampicillin); 8.20% to CEFT (Ceftiofur); 6.56% to ENRO (Enrofloxacin); 14.75% to FLU (Florfenicol); 4.92% to GENT (Gentamicin); 1.64% to NEO (Neomycin); 29.51% to OXY (Oxytetracycline); 26.23% to TET (Tetracycline); and 1.64% to SXT (Trimethoprim–sulfamethoxazole). Additionally, intermediate resistance was detected in 1.64, 4.92, 3.28, and 3.28% of isolates for CEFT, ENRO, FLU, and TET, respectively (Figure 6A). Under anaerobic conditions, the following resistance rates were observed: 11.48% of the isolates were resistant to AMP (Ampicillin); 8.20% to CEFT (Ceftiofur); 6.56% to ENRO (Enrofloxacin); 11.48% to FLU (Florfenicol); 32.79% to GENT (Gentamicin); 39.34% to NEO (Neomycin); 29.51% to OXY (Oxytetracycline); 8.20% to TET (Tetracycline); and 3.28% to SXT (Trimethoprim–sulfamethoxazole). Additionally, intermediate resistance was detected in 4.92, 1.64, 4.92, 60.66, 57.38, 1.64, 1.64, 21.31, and 6.56% of isolates for AMP, CEFT, ENRO, GENT, NEO, OXY, SXT, TET, and FLU, respectively (Figure 6B). The CLSI veterinary guidelines do not specify ZOI breakpoints for Florfenicol + Flunixin meglumine (FF), Gamithromycin (GAM), Penicillin (PEN), and Tulathromycin (TUL) in the context of *E. coli*. Consequently, it is not possible to categorize these antimicrobials as resistant, intermediate, or susceptible. To aid in interpretation, Figure 7 presents a boxplot depicting the mean ZOI values for all tested drugs under both aerobic and anaerobic conditions. Based on clinical breakpoints for the order Enterobacterales, 30% (18/60) of the *E. coli* isolates displayed AMR to no less than one drug tested under aerobic conditions, while 68% (41/60) showed resistance under anaerobic conditions. In contrast, nine *E. coli* isolates (15%) exhibited an MDR phenotype under both aerobic and anaerobic conditions (Figures 8A,B).

Notably, all isolates exhibiting phenotypic resistance to at least one tested antimicrobial belonged to the B1 phylogroup. However, none of the tested factors—phylogroup, isolate, or farm—had a significant effect on phenotypic resistance counts (*p*-value > 0.05).

### 3.4 Comparison between antimicrobial susceptibility genotypes and phenotypes

To assess the potential of WGS in predicting AMR profiles, we compared genotypic data (gene presence) with phenotypic resistance behaviors. Understanding the concordance between these two factors is essential for evaluating the accuracy of genetic predictions in reflecting observed resistance patterns. For instance, a strong agreement (Kappa > 0.80) was observed between genotype-based resistance (presence of *tetA*, *tetB*, or *tetD* genes) and phenotypic resistance (determined by a ZOI diameter ≤10 mm) for both tetracyclines tested (tetracycline and oxytetracycline). This finding is particularly important, as *E. coli* exhibiting high concordance between genotype and phenotype could be utilized in AMR surveillance programs, where gene presence is strongly correlated with the resistant phenotype. However, the comparison between genotypic and phenotypic AMR profiles can be complex due to the varying resistance mechanisms across different antimicrobial agents and bacterial species. While the genotype represents the genetic “potential” for resistance, it does not always correspond fully to the phenotype expressed by the bacterium. The consonance between genotypic and phenotypic drug resistance was good for AMP (kappa = 0.762) and very good for GENT (kappa = 1), TET (kappa = 0.875), OXY (kappa = 0.96), and SXT (kappa = 1), as demonstrated in Table 2. In contrast, CEFT (kappa = 0), ENRO (kappa = −0.0274) displayed poor agreement, while FLU (kappa = 0.36) demonstrated fair agreement and NEO (kappa = 0.487) showed moderate agreement (Table 2).

**Table 2:** Contingency table comparing genotypic resistance to phenotype resistance.

Antimicrobial	Susceptible phenotype		Resistant phenotype		Kappa coefficient	Agreement	*p*-value
	Resistant genotype	Susceptible genotype	Resistant genotype	Susceptible genotype			
AMP	4	48	8	0	0.762	Good	1.23e-09
CEFT	12	48	0	0	0	Poor	–
FLU	6	52	2	0	0.366	Fair	2.45e-04
NEO	2	57	1	0	0.487	Moderate	1.1e-05
GENT	0	57	3	0	1	Very good	9.55e-15
ENRO	1	55	0	4	−0.0274	Poor	0.788
TET	3	42	15	0	0.875	Very good	8.41e-12
OXY	1	42	17	0	0.96	Very good	1.01e-13
SXT	0	59	1	0	1	Very good	9.55e-15

AMP, Ampicillin; CEFT, Ceftiofur; FLU, Florfenicol; NEO, Neomycin; GENT, Gentamicin; ENRO, Enrofloxacin; TET, Tetracycline; OXY, Oxytetracycline; SXT, Trimethoprim–sulfamethoxazole.

## 4 Discussion

### 4.1 Resistome profiling and phylogroup distribution in *Escherichia coli* from Florida farmed WTD

In this study, whole genome sequencing of *E. coli* genomic DNA from various tissue samples of WTD was employed to quantify ARGs and report the relative abundance of the various genetic determinants attributed to AMR. A total of 362 distinct ARGs were identified, conferring resistance to 12 antimicrobial classes through 19 different mechanisms. These results exceed those of similar studies using different matrices, such as roe deer feces (41 ARGs from 7 antimicrobial classes) (Dias et al., 2022), soils treated with bovid and swine manure (77 ARGs from 8 antimicrobial classes) (Chen et al., 2019), and porcine excrement (146 ARGs from 9 antimicrobial classes) (Zhao R. et al., 2018; Zhao Y. et al., 2018).

In our analysis of *E. coli* isolated from WTD tissue samples, we identified a range of ARGs that align with documented antimicrobial resistance trends across wildlife and agricultural species. Two classes of the core resistome, MLS and MDR, are especially concerning as these classes are less specific and are responsible for conferring resistance to numerous antimicrobials or classes. Overall, ARGs associated with β-lactam resistance were highly abundant, along with those conferring MDR and resistance to individual classes like bacitracin, cationic antimicrobial peptides (CAP), macrolide-lincosamide-streptogramin (MLS), aminoglycoside, and tetracycline. These antimicrobial classes mirror those commonly used in global livestock production systems (He et al., 2020) as well as ice core samples without anthropogenic influence (Paun et al., 2021). Their widespread presence has also been documented in wildlife, particularly near anthropogenic sources such as manure and biosolid application sites. For example, tetracycline resistance genes (*tetQ*) were frequently detected in wild deer in such environments, highlighting the impact of human activity on wildlife resistomes (Rogers et al., 2018). Comparable ARG profiles have been observed in farmed sika deer in China, where tetracycline resistance genes were similarly predominant (Huang et al., 2016), and in wildlife gut microbiota across species in Poland, where *tetQ* was the most prevalent ARG (Skarżyńska et al., 2020).

Our findings also corroborate above mentioned patterns, with *mphB*—an ARG conferring resistance to MLS—identified in over 98% (59/60) of the samples. The highest relative abundance of *mphB* was observed in samples S11 and S30, where it represented 13.8% (551/527,233) and 11.5% (229/52,733) of total reads, respectively. Additionally, *floR*, associated with chloramphenicol and florfenicol resistance (White et al., 2000), exhibited the highest relative abundance among all samples and ARG groups, reaching 16.3% (4,122/32,389). This prevalence echoes findings in cattle farming where, β-lactam, MLS, aminoglycoside, and tetracycline ARGs to be dominant (Muurinen et al., 2017; Qian et al., 2018; Zhao R. et al., 2018; Zhao Y. et al., 2018; Chen et al., 2019; Wu et al., 2020). Recent studies have also reported a rising prevalence of β-lactam resistance, including cephalosporin- and carbapenem-resistant genotypes, highlighting the widespread dissemination of these ARGs in companion animals, dairy cattle, and wastewater (Daniels et al., 2018; Mollenkopf et al., 2018; Mathys et al., 2019). This trend is also consistent with a linear increase in β-lactam resistance, including cephalosporins, observed in *E. coli* from WTD in Ohio, United States (Ballash et al., 2021).

Beyond our specific findings, we identified ARGs such as *sulII* (sulfonamide resistance), *qnrS* (fluoroquinolone resistance), *blaTEM* (class A β-lactamase), and various *aph* genes (aminoglycoside O-phosphotransferases), which are recognized as environmental resistance markers (Berendonk et al., 2015). These findings reveal a complex ARG profile in farmed WTD from Florida, influenced by both environmental factors and human activities related to antimicrobial resistance. This study is the first to characterize the resistome of farmed WTD in Florida, identifying five high-risk antimicrobial resistance genes (ARGs) present at relatively high abundances. These ARGs are considered ‘present hazards’ due to their association with ESKAPE pathogens—*Enterococcus faecium*, *Staphylococcus aureus*, *Klebsiella pneumoniae*, *Acinetobacter baumannii*, *Pseudomonas aeruginosa*, and *Enterobacter* species—which pose a global threat to human health and their potential for horizontal gene transfer (HGT) (Zhang et al., 2021). These insights provide valuable guidance for managers, farm owners, and veterinarians, supporting informed decisions on medication use in Florida deer farms.

The distribution of *E. coli* phylogroups observed in this study aligns with prior research (Touchon et al., 2020; Munkhdelger et al., 2017). The most prevalent phylogroups were B1, A, and D, while C, E, and F were the least common. Phylogroups A and B1, commonly linked to commensal strains, are generally less virulent but exhibit notable AMR in specific contexts, particularly in agricultural environments. These phylogroups often harbor ARGs for tetracyclines, sulfonamides, and aminoglycosides (Monroy-Pérez et al., 2020; Pais et al., 2022). Although phylogroup A showed an overall lack of resistance, ARGs for tetracyclines, sulfonamides, and aminoglycosides were detected in phylogroup B1. In our study, phylogroup B1 is predominant and demonstrates high resistance to tetracyclines, β-lactams, and sulfonamides and MDR, which is lower than in clinical isolates (Pais et al., 2022). While the prevalence of MDR and ESBL genes were lower than in clinical isolates, their presence still poses a significant risk of zoonotic transmission (Pais et al., 2022).

Interestingly, although phylogroup D was the third most prevalent group in this study, it accounted for only 0.05% (3/60) of total isolates—a markedly lower proportion than the 26 and 28.4% reported in previous studies in humans, domestic and wild animals and the environment (Touchon et al., 2020; Munkhdelger et al., 2017). Phylogroup D is generally considered more virulent than phylogroups A and B1, typically associated with extraintestinal pathogenic *E. coli* (ExPEC) in humans rather than with commensal strains (Clermont et al., 2000; Da Silva and Mendonça, 2012; Picard et al., 1999).

A notable finding in the present study was that only one isolate (S36) belonged to phylogroup B2, a strikingly low proportion compared to the 39 and 33.8% reported by Touchon et al. (2020) and Munkhdelger et al. (2017), respectively. This discrepancy is surprising given the dominance of phylogroup B2 in isolates from Asia (Zhao et al., 2015; Lee et al., 2016; Bashir et al., 2012), Europe (Ejrnæs et al., 2011; Dubois et al., 2010), Africa (Dadi et al., 2020), and North America (Paniagua-Contreras et al., 2017). In these studies, B2 isolates frequently harbored ESBL genes and MDR profiles, particularly against cephalosporins and fluoroquinolones (Monroy-Pérez et al., 2020; Hemati et al., 2024). In contrast, no resistance determinants were identified in the phylogroup B2 isolate from our study. These differences highlight the variability in phylogroup distribution across geographic regions and underscore the need for further investigation to understand the factors driving these patterns.

### 4.2 AMR profiles of *Escherichia coli* isolated from farmed WTD in Florida

The bacterial AMR profile of various vertebrate species and the environment is still primarily assessed through culture-dependent methods (Guitor et al., 2019). In this study, we assessed the prevalence of AMR by evaluating the susceptibility of *E. coli* isolated from farmed WTD in Florida to 14 commonly used farm-associated antimicrobials. Based on clinical breakpoints for the family *Enterobacteriaceae*, 30% (18/60) of the *E. coli* isolates displayed resistance under aerobic conditions, while 68% (41/60) showed resistance under anaerobic conditions. These values exceed the resistance rates reported in venison from Germany (9%) and red deer in Spain (7, 23%) (Mateus-Vargas et al., 2017; Alonso et al., 2016; Dias et al., 2022). In the U. S., 16.7% of *E. coli* isolates obtained from bison (*Bison bison*) carcasses exhibited resistance to at least one antimicrobial agent (Li et al., 2007).

In our study, *E. coli* isolates predominantly exhibited phenotypic resistance to tetracyclines under aerobic conditions. A high proportion of isolates also demonstrated phenotypic resistance to penicillin when interpreted using ZOI breakpoints reported in previous studies (Bughe et al., 2020). However, as the CLSI does not provide breakpoints for penicillin in *E. coli,* we excluded penicillin from formal phenotypic categorization. This observation is consistent with the findings of wild roe deer (Mayrhofer et al., 2006) and farmed red deer (Alonso et al., 2016), but contrasts with those of wild red deer (Dias et al., 2022) and wild WTD (Ballash et al., 2021), where resistance to ß-lactams and sulfonamides was most prevalent. The *tetA* and *tetB* gene groups, responsible for encoding an active efflux pump, were present in all isolates with phenotypic resistance to tetracyclines, indicating these gene groups play a key role in tetracycline resistance observed in our study. Under anaerobic conditions, aminoglycosides, specifically GENT and NEO, demonstrated the highest resistance rates, with 32.8 and 39.3%, respectively. This is expected, as aminoglycosides require oxygen to cross the cell membrane (Whelton, 1984), and the accumulation of reactive metabolic byproducts has been noted in cells treated with these antibiotics (Wong et al., 2022). Penicillin was included in the AST, despite the lack of ZOI breakpoints for the medication in the CLSI VET01S. It is important to note that if the ZOI breakpoints from previous studies were applied for resistant, intermediate, or susceptible categorization, 95% (57/60) of isolates would have been resistant to penicillin under aerobic conditions (Bughe et al., 2020).

In the present study, isolates exhibiting phenotypic resistance to three or more antimicrobials were considered MDR, following the criteria by Begum et al. (2018). Under aerobic conditions, nine *E. coli* isolates (15%) exhibited a MDR phenotype, a significantly higher proportion compared to farmed red deer (1/72, 1.3%) (Alonso et al., 2016), wild red deer (2/101, 1.9%) (Dias et al., 2022), and wild roe deer (1/76, 1.3%) (Mayrhofer et al., 2006). Similarly, under anaerobic conditions, nine *E. coli* isolates (15%) also exhibited a MDR phenotype. The high prevalence of multidrug-resistant (MDR) strains may be attributed to the indiscriminate use of antimicrobial agents (Van Den Bogaard and Stobberingh, 2000). Nevertheless, the MDR patterns identified in this study offer valuable insights for farm managers, owners, and veterinarians in selecting appropriate treatments for use in deer farms—for instance, by choosing antimicrobials that retain efficacy and avoiding those associated with high resistance rates.

Our findings underscore the advantages of utilizing both culture-dependent and culture-independent methods to investigate drug resistant bacteria, as they complement each other. For example, ß-lactam resistance-associated ARGs were the most abundant across all samples, yet only seven of the isolates showed phenotypic resistance to two antimicrobials commonly used on deer farms: CEFT and AMP. Moreover, the presence of gene groups: *cmy*, *ctx*, or *tem* could serve as a proxy for phenotypic resistance for AMP, which exhibited good agreement (kappa = 0.762), but not for CEFT (kappa = 0). However, the presence of ARGS in our analysis was in good agreement with an intermediate phenotype for CEFT. In addition, *tetA*, *tetB*, or *tetD* gene groups showed very good agreement with the phenotypic resistance in isolates from farmed WTD in Florida. These findings are encouraging, especially as tetracyclines are among the most widely used antimicrobial classes in veterinary (Daghrir and Drogui, 2013) and agricultural production (Chang et al., 2023). Despite the challenges in drawing direct comparisons, both approaches need globally standardized methodologies. Software like the AMR++ pipeline, coupled with the ResistoXplorer platform, performed exceptionally well in standardizing metagenomics-based results, despite the diversity of upstream approaches.

The results of this study showed that none of the evaluated factors—phylogroup, isolate, or farm—significantly affected phenotypic resistance counts (*p* > 0.05). Thus, no associations were detected between resistance phenotypes and either genetic background or environmental origin within the dataset. Nevertheless, the observation that phenotypic resistance occurred exclusively in phylogroup B1 suggests an intriguing trend that merits further investigation.

While our study provides valuable information on resistance profiles from Florida’s farmed WTD, it is important to consider the limitations of our study design. The overrepresentation of phylogroups A and B1 in the dataset may have introduced bias, potentially underestimating the role of other phylogroups, such as B2 and D, which are known to frequently harbor multidrug-resistant strains (Hemati et al., 2024; Tohmaz et al., 2022). Phylogroup B1, commonly associated with commensal strains, has also been linked to resistance determinants, especially in agricultural and environmental settings. The diverse resistance profiles observed within this phylogroup highlight its adaptability to selective pressures, such as the use of antibiotics in livestock (Hemati et al., 2024; Raimondi et al., 2019). The absence of significant associations might also be explained by a limited sample size or insufficient diversity in metadata factors. Future studies with a more balanced representation of phylogroups and broader environmental and clinical contexts may uncover nuanced relationships between genetic background and resistance patterns. This underscores the importance of expanding datasets to capture a more comprehensive view of how resistance determinants are distributed across *E. coli* populations.

This study has several limitations. First, the samples were obtained from necropsied farmed WTD, which may not fully represent the broader farmed or wild deer populations across Florida. The use of *E. coli* as an indicator organism, while informative, captures only the culturable fraction of the microbial community and may overlook additional ARGs present in unculturable taxa. Although whole-genome sequencing provided valuable genotypic insights, it has limited ability to resolve mobile genetic elements such as plasmids and transposons, which are central to AMR dissemination. Furthermore, discrepancies between genotypic predictions and phenotypic resistance highlight the need for functional validation beyond genomic data. Finally, the cross-sectional design and lack of detailed antimicrobial usage records restricted our ability to assess temporal dynamics or directly link farm management practices with resistance outcomes.

## 5 Conclusion and future directions

The detection of ARGs in *E. coli* isolated from farmed WTD in Florida underscores the presence of resistance to multiple antimicrobial classes. In total, 362 unique ARGs were identified, conferring resistance to 12 antimicrobial classes through 19 distinct mechanisms. Among the *E. coli* isolates, 30% (18/60) exhibited resistance to at least one antimicrobial agent under aerobic conditions, while 15% (9/60) demonstrated a MDR phenotype. Notably, five high-risk ARG groups—*aac3*, *aph6*, *floR*, *mphA*, and *mphB*—were found in high abundance, with *mphB* and *floR* particularly prevalent, comprising up to 13.8 and 16.3% of total ARG reads, respectively.

These findings highlight an urgent need for targeted management practices to limit disease transmission and mitigate the development and spread of antimicrobial resistance. Certain ARGs may serve as effective bioindicators for environmental health monitoring, resistance quantification, strategy evaluation, and predictive modeling of ARG distribution (Ishii, 2020). To address these risks, robust compartmentalization practices—including the implementation of barrier fencing—are critical for preventing direct and indirect transmission of ARGs between farmed WTD and surrounding free-ranging populations.

The high prevalence of resistance genes—particularly those associated with β-lactam antibiotics (49%) and MDR phenotypes (14.8%)—emphasizes the potential for horizontal gene transfer between wildlife and livestock. Routine surveillance of ARGs and pathogenic organisms in farmed WTD is essential to guide antimicrobial stewardship and anticipate emerging resistance trends. Monitoring key indicator genes such as *sulI*, *sulII*, *aadA*, *bacA*, *oqxA*, *ermB*, and *mexE* may provide early warnings of ARG proliferation (Zhao R. et al., 2018; Zhao Y. et al., 2018; Tarek and Garner, 2023).

Tracking the progression of resistance, particularly to drugs like ceftiofur and enrofloxacin—both associated with intermediate resistance phenotypes—can inform timely updates to treatment protocols. This study also confirmed strong genotype–phenotype agreement for several resistance genes, such as *cmy*, *ctx*, and *tem* for ampicillin; *tetA*, *tetB*, and *tetD* for tetracycline and oxytetracycline; *aac3* for gentamicin and neomycin; and *sulII*, *sulIII*, and *dfrA* for sulfamethoxazole-trimethoprim. These genes should be prioritized in resistance monitoring due to their predictive power for phenotypic resistance.

Antimicrobial treatment in farmed WTD should be tailored to resistance profiles to ensure therapeutic efficacy and manage potential co-infections. Drugs with low genotype–phenotype agreement—such as ceftiofur, florfenicol, and enrofloxacin—may be more suitable for targeted use, whereas antimicrobials with high resistance rates and strong genotype–phenotype agreement (e.g., tetracycline, oxytetracycline, and ampicillin) should be avoided. Tetracycline and oxytetracycline exhibited the highest rates of resistance (26.2 and 29.5%, respectively), making them unsuitable for prophylactic or routine treatment on Florida’s WTD farms.

Although the absence of ZOI breakpoints for penicillin in the CLSI manual limited definitive conclusions, reference to prior literature suggests that approximately 95% of isolates would be classified as resistant—further supporting its exclusion from treatment protocols (Bughe et al., 2020). In summary, farmed WTD may serve as reservoirs for ARGs with the potential to impact both agricultural and ecological systems. By integrating ARG surveillance, antimicrobial susceptibility data, and tailored management strategies, stakeholders can more effectively control disease spread, limit resistance development, and safeguard both animal and environmental health.

## Data Availability

The original contributions presented in the study are publicly available. This data can be found here: https://www.ncbi.nlm.nih.gov/PRJNA1183872.
